# Cognitive rehabilitation of neuropsychological deficits and mild cognitive impairment: a review of the literature

**DOI:** 10.1590/S1980-57642009DN20200011

**Published:** 2008

**Authors:** Eliane Correa Miotto, Valéria Trunkl Serrao, Gláucia Benutte Guerra, Mara Cristina Souza de Lúcia, Milberto Scaff

**Affiliations:** 1 Clinical Director of the Division of Psychology, Hospital das Clinicas, University of São Paulo, Brazil.; 2 Post-Graduate Student in Clinical Neuropsychology, Hospital das Clinicas, University of São Paulo, Brazil.; 3 Research Director of the Division of Psychology, Hospital das Clinicas, University of São Paulo, Brazil.; 4 Director of the Psychology Division, Hospital das Clínicas, University of São Paulo, Brazil;; 5 Professor of the Neurology Department, Hospital das Clínicas, University of São Paulo, Brazil.

**Keywords:** mild cognitive impairment, neuropsychological rehabilitation, cognitive training

## Abstract

Neuropsychological rehabilitation is related to the treatment or optimization of disabilities, handicaps and cognitive deficiencies including emotional, behavioral and personality alterations, aiming at the best cognitive, neurobiological and social re-adaptation.

**Objective:**

The main aim of this paper is to review scientific studies published over the last five years on cognitive training with rehabilitation, focusing on elderly subjects with cognitive complaints and patients diagnosed with MCI.

**Methods:**

Data were generated from *Medline, PsychoInfo* and *EMBASE* including publications from 2002 to 2007 using the search terms “Mild Cognitive Impairment”, “Cognitive Complaints”, ”Rehabilitation” and “Intervention Studies”. Data collection criteria were restricted to the quality of evidence Class I.

**Results:**

Eight articles out of sixty eight previously selected were chosen because of their randomized studies, including techniques of cognitive rehabilitation in patients with cognitive complaints, MCI and neuropsychological training.

**Conclusions:**

The studies showing generalization of rehabilitation techniques to practical real life situations and use of an errorless learning approach were considered more effective in terms of maintaining treatment follow up, although further studies are recommended.

## Abstract (pt)

A reabilitação cognitiva se ocupa do tratamento e otimização das incapacidades, desvantagens e deficiências cognitivas, bem como de alterações emocionais, comportamentais e de personalidade. Visa a melhor readaptação neuropsicológica, neurobiológica e social.

**Objetivo:**

Realizar revisão literária sobre os estudos científicos publicados nos últimos cinco anos em reabilitação cognitiva, em sujeitos com queixas cognitivas e pacientes idosos diagnosticados com TCL.

**Métodos:**

As informações foram obtidas nas bases de dados *Mediline, PsychoInfo* e *EMBASE* com recomendações baseadas na qualidade de evidência Classe I.

**Resultados:**

Foram encontrados 68 artigos. Destes, selecionados oito, por caracterizarem estudos randomizados, incluir técnicas de reabilitação cognitiva em pacientes com queixas cognitivas e TCL e por documentarem avaliações neuropsicológicas.

**Conclusões:**

As pesquisas que demonstraram técnicas de reabilitação com abordagens capazes de generalização para as atividades práticas da vida e uso da abordagem aprendizagem sem erro se mostraram mais efetivas quanto à manutenção no seguimento, contudo, são necessários estudos mais detalhados.

Due to the growth in the elderly population over recent decades, an increase in the number of degenerative diseases including dementia has been documented. Consequently, an increasing number of non-pharmacological treatments aimed at cognitive improvement have emerged,^1^ particularly because these deficits are the first to appear, affecting memory, behavior alterations and loss of functional activities.^2^

Mild Cognitive Impairment (MCI)^3^ has been widely studied in the international literature^4^ as a response to the aging global population. The international Working Group in Mild Cognitive Impairment^5^ recommends the assessment of complex instrumental activities and a variety of cognitive functions including memory, attention, executive functions, praxis, language and visuo-perception abilities for differential diagnosis. In particular, an objective memory impairment and/or other cognitive impairment must also be present to characterize MCI.^6^ This decline should be confirmed by the person, family, and by neuropsychological evaluation which enables cognitive alterations to be identified at a very early stage.

Several studies have reported that the earlier the identification of the MCI subtype and beginning of therapy, the better the results concerning the improvement in cognition.^7,8^ The clinical and pharmacological interests have grown because of the high possibility of the development of dementia. In an attempt to better understand this process^9^ some practical guidelines have been proposed by some studies.^10^

A Canadian study^11^ performed over a period of five years, showed that the prevalence of MCI varied between 1.03 to 3.02%, with the criteria of loss of memory or impaired daily activities not being used. There was an increase in the risk of death, institutionalization and development of dementia (mainly Alzheimer Disease – A.D.) at the end of follow up, demonstrating the importance of evaluating and monitoring patients with MCI.^12^ The annual conversion rate to A.D. was approximately 10 to 15%, representing an increased subgroup to develop this dementia syndrome, particularly those who presented MCI of multiple domains.^13^

Additional exams for diagnosis such as neuroimaging exams are not yet specific for A.D. and have not been validated in MCI detection. Discussions on this issue remain pertinent since the concept of MCI has only recently been and neuropathological data are limited.^14^

Since there is no cure or reversal of neurological deterioration caused by dementia, the available pharmacological and non-pharmacological treatments try to minimize the speed of progression of the symptoms, especially those related to memory difficulties. Among non-pharmacological treatments, the rehabilitation of cognitive deficits or neuropsychological rehabilitation are highlighted.^9,14-18^

Most neuropsychological interventions include the treatment or adjustment of emotional and behavioral alterations in addition to cognitive, neurobiological and social readaptation.^19^ In this context, a broad comprehension of the concept and objectives of neuropsychological rehabilitation should include the following aspects:^20^

(1) rehabilitation conducted together with the patients with brain lesion, their family members and a multi-professional team;
(2) before any rehabilitation program is started it is necessary to identify realistic goals of treatment;
(3) the neuropsychological interventional programs should address the cognitive deficits, as well as the emotional and psychological problems;
(4) the compensation of difficulties have been widely benefited by technological advance;
(5) the rehabilitation treatment should go beyond the outpatient clinic or private consulting room and address real life difficulties in ‘real environments’;
(6) like any other area of study, neuropsychological rehabilitation needs a theoretical base that includes models and methods from different areas.

Over the past thirty years, the literature has described a number of memory rehabilitation techniques, including compensatory and restorative techniques. However, there are few neuropsychological techniques based on theoretical models. One such model, Goal Management Training (GMT) developed by Robertson (1996)^21^ was derived from theories of executive function particularly from Duncan’s (1995)^22^ theory of goal neglect. In this technique, patients are trained to ‘stop and think’ about various problems and goals before and during the performance of a task. They are also trained to use a ‘mental blackboard’ or working memory to keep track of what they are doing and check if it matches their original goal.

Another technique, Errorless Learning (EL), prevents people from making mistakes when learning new information or a new skill. This procedure is based on the notion that without proper declarative memory that enables one to remember and then correct errors or learning through trial and error, error prevention should be adopted (Baddeley & Wilson, 1994).^23^ It has been suggested that errorless learning works through implicit memory, normally preserved in severe memory disorders and in amnesics. In cases of partial preservation of episodic memory, it works by strengthening explicit memory.

Recently, a number of studies have included computerized cognitive training (CCT) in their neuropsychological rehabilitation program aimed at stimulating specific cognitive functions such as memory, attention, language or executive functions. These computer programs include Smartbrain (www.educamigos.com), the Training Neuropsicológico di Mario (TNP) software^24,25^ used to stimulate specific cognitive functions^26^ and other programs still undergoing investigation.

Various studies using cognitive training focusing on memory, attention, language or executive deficits^27^ have suggested that it is possible to improve some of these cognitive deficits.^28^ However, only a few studies addressed the rehabilitation of these cognitive deficits in elderly patients with MCI and cognitive deficits. This article aimed to perform a literature review over the last five years on cognitive training in elderly patients with cognitive complaints or diagnosed with MCI.

## Methods

The main databases used were Ovid-Medline, PsychINFO and EMBASE including publications from 2002 to 2007. The terms “MCI, Cognitive Deficits, Memory Complaint, Neuropsychological Rehabilitation, Cognitive Training and Cognitive Rehabilitation” were included as main descriptors, in Portuguese or English. The selected publications were restricted to the quality of evidence Class I – studies using randomized and controlled groups. Class II – non-randomized and non-controlled group studies and class III – case studies were not included.^29^ According to guidelines from the American Academy of Neurology, “Class I studies have a lower risk of bias than class II or III studies”.^29,30^

## Results

Amongst the 68 articles found, eight were selected and are characterized in Table 1. These are randomized controlled studies with techniques of cognitive rehabilitation in elderly subjects with memory complaints or MCI.

**Table 1:** Cognitive training interventions: article summary data.

#	Study	Methods	Participants	Assessment battery
1	Winocur et al., 2007^33^	12 weeks long and conducted in a small-group for­mat provided comprehensive training in three dis­tinct but integrated modules: memory, modified Goal Management, and Psychosocial function. The experimental design consisted of two groups, each receiving the same treatment and control procedures, according to a multiple baseline, crossover design. Early or late. Training group was completed in a blocked quasi-random format.	Participants in the trial were between 71 and 87 years of age. (N=49)	Logical Stories Test; Hopkins Verbal Learning Test; Self-assessment questionnaire - (SAQ).
2	Talassi et al., 2007^34^	Compared two treatments: a cognitive rehabilitation program (experimental) and non-cognitive rehabilita­tion program (control). Cognitive program: comput­erized cognitive training (CCT), occupational therapy (OT) and behavioral training (BT). The control group consisting of Physical rehabilitation (PR), OT and BT. Both programs provided 30-40 minute sessions held for every activity, on 4 days per week, covered a 3-week-period. They compared the results from base­line and post-treatment performance from the two conditions, namely the experimental treatment and the control treatment group of community-dwell­ing subjects with MCI and MD (Mild Dementia).	Experimental MCI group (n=30) and Control MCI group (n=7). Experi­mental MD group (n=24) and Con­trol MD group (n=5).	Mini Mental State Examination (30-item); Forward and backward digit span; Phonemic and semantic verbal fluency; Sub-test for episodic memory of Riv­ermead behavioral memory test; Visual Search; Digit Symbol test; Rey complex figure copy and recall, and Clock-drawing test. Geriatric Depression Scale (GDS-30); State-trait anxiety inventory (Stai-Y1 and Stai-Y2) and Neuropsychiatric inventory (NPI). Physical Perfor­mance test (PPT), Basic ADL (BADL) and Instrumental ADL (IADL). Caregiver burden inventory (CBI).
3	Cipriani et al., 2006^26^	Each person attended two training programs. A single training program (13-45 min sessions held on 4 days per week) covered a 4-week-period. The break between the first and the second training program lasted 6±2 weeks. The design consisted of individual­ized rehabilitative intervention. The baseline results were compared with those collected at the 3-months follow-up. Statistical analysis of the differences was performed with non-parametric Wilcoxon test, using SPSS 10.0 software.	Alzheimer disease (n=10) - aged 74.1±5.6 years MCI (n=10) aged 10.6±6.0 years; Multiple System At­rophy - MAS (n=3) aged 69.0±9.5 years were selected from the same setting in order to have a different control group.	MMSE and the following tests: phonemic and seman­tic verbal fluency (cued verbal production); visual search (sustained attention), trail making test A/B (vi­sual search, attention and executive functions); digit symbol test (psychomotor learning), and Rivermead behavioral memory test (behavioral memory). GDS; Advanced activity of daily living (AADL); State anxiety (STAI-X1) and trait anxiety (STAI-X2) and short form health survey (SF-12)
4	Akhtar et al., 2006^35^	Volunteers participated individually in an experi­mental session that lasted 40-60 minutes. They were instructed that they would be learning two lists of 10 words in errorless and errorful learning conditions. Was adopted a meta-cognitive approach measuring people's memory monitoring through judgments of learning (JOLs) a prediction of feature memory performance. The experiment considered a within subject design.	MCI (n=16); (health) older adults group - OAC (n=16). MCI patients attended a memory clinic and the OAC were volunteers who were com­munity dwelling.	CAMCOG, MMSE, NART, CERAD (word-learning list).
5	Ball et al., 2002^37^	Conducted in small group setting in ten 60 to 75 minute sessions over 5 to 6 week periods (Behavioral interventions with no-pharmacological component). In all three conditions, sessions 1 through 5 focused on strategy instruction and individual and group ex­ercises to practice the strategy. Sessions 6 through 10 provided additional practice exercises but introduced no new strategies. This was a randomized, controlled, single blind trial.	Volunteer sample of 2832 persons aged 65 to 94 years recruited from senior housing, community centers, and hospital/clinics in 6 metropolitan areas in the USA.	MMSE; SF-36 physical function.
6	Ball et al., 2007^36^	Across studies, participants included community-dwelling older adults ranging in age from 55 to 95 years (M=73.94 SD=5.96). This was a randomized study.	Across studies, participants included 2,039 community-dwellers.	Useful Field of View Test (UFOV); Digit Symbol copy; Letter comparison; Patter comparison; Stroop; Trails B; Road Sign test; Rey-O Immediate Memory; Benton Visual Retention and Pelli-Robson Contrast Sensitivity.
7	Levine et al., 2007^31^	Modified GMT for each module lasted 4 weeks. Each group received the intervention immediately and the other was on waiting list prior to rehabilitation. This was a crossover design study.	46 community-dwelling adults 71-87 years of age were recruited from advertisements and word of mouth. They were quasi-randomly assigned to an Early Training Group (ETG n= 26) and a Late Training Group (LTG n=20). Mean age (for both groups) M=79; SD=2.4 and 6.4 (for the ETG and LTG, respectively).	Simulated real life tasks (SRLTs) and Dysexecutive questionnaire (DEX).
8	Rozziniet al., 2007^38^	One year study period. At both evaluations at base­line and after one year follow-up cognitive tests were administered in approximately one hour session. Neuropsychological rehabilitation (TNP software) has been modulated on complexity input/output modalities and length. This was a longitudinal and retrospective study.	Subjects with MCI (n=59). Subjects (n=50) were randomized to receive TNP plus Cholinesterase inhibitors alone and subjects (n=22) no treat­ment.	MMSE; short story recall; category fluency and letter fluency; Raven's colored matrices; copy and delayed re­call of Rey's figure; neuropsychiatric inventory (NPI) and GDS, BADL and IADL.
1	Winocur et al., 2007^33^	Simulated real-life tasks (SRLTs) in modi­fied Goal Management (GMT)	Memory measures (Logical Stories Test) revealed substantial improvement for immediate (n2=.18) and delayed recall (n2=.10). In the HVLT, the benefits were more modest in subject organization (n2=.16), category clustering (n2=.09) and secondary memory (n2=.08). GMT substantially improved following training. Effect size for the overall GMT score (n2=.24) and is related measures of task strategy (n2=.30) and checking (n2=.19) were high. For the GMT engagement score, the effect size could be considered medium (n2=.12). The SAQ, results indicated that both groups believed they were leading more meaning­ful lives (n2=.18), that their memories were better (n2=.35) and that they were better at setting and achiev­ing practical goals (n2=.23)	
2	Talassi et al., 2007^34^	Computer-based cognitive training.	MCI group obtained a significant improvement in Figure Rey copy (p=0.033) and PPT (p=0.003). Symp­toms of depression and anxiety showed a significant reduction (GDS: p=0.012; STAI-Y1: p=0.030; STAI-Y2: p=0.000). MD group showed a significant improvement in global cognitive status (MMSE: p=0.002), and a significant reduction of depression and anxiety symptoms (GDS: p=0.030; STAI-Y1: p=0.011; STAI-Y2: p=0.044). In MD group performing control program only a significant improvement of score in the test of semantic verbal fluency (p=0.43) was observed at the end of the treatment.	
3	Cipriani et al., 2006^26^	Software for neuropsychological training (attention, memory, perception, visuospa­tial cognition, language and non-verbal intelligence).	The AD group showed a significant MMSE score improvement (p=0.010). MMSE scores at baseline and a follow-up remained quite stable in the other two groups. AD patients also showed significant improvement in the areas of verbal production (p=0.036). MCI patients obtained a significant improvement in behavioral memory (p=0.017; p=0.011). No significant improvement was observed in MAS group.	
4	Akhtar et al., 2006^35^	Errorless learning (EL) and Errorful learn­ing (EF).	The results revealed errorless learning is an effective memory rehabilitation tool for people with MCI, with significant increases in recall performance for both groups relative to errorful learning. Participants were aware of the benefits of errorless learning in their JOLs (both, MCI and Control).	
5	Ball et al., 2002^37^	ACTIVE - Advanced Cognitive Training for independent and Vital Elderly) were included 3 distinct cognitive interventions: memory, reasoning and speed training.	Each intervention improved the targeted cognitive ability compared with baseline, lasting for 2 years (P< .001 for all). 87% of speed; 74% of reasoning and 26% of memory-trained participants demonstrated reli­able cognitive improvement immediately after the intervention period. Booster training enhanced training gains in speed (P<. 001) and reasoning (P<. 001 for both). No training effects on everyday functioning were detected at 2 years.	
6	Ball et al., 2007^36^	Useful Field of View (UFOV)	Results indicated that training produces immediate improvements across all subtests of the UFOV, par­ticularly for older adults with initial speed of processing deficits (p<.001).	
7	Levine et al., 2007^31^	Modified GMT (included memory skills training and Psychosocial Training modules).	Results indicated improvements in SRLT performance and self-rated executive deficits coinciding with the training in both groups. These gains were maintained at long-term follow-up. Training protocol de­signed to increase real-life goal attainment through interactive, task-based training in attention control and self-organization.	
8	Rozziniet al., 2007^38^	Multidimensional software (TNP software) Multidimensional software covered differ­ent cognitive functions such as memory, reasoning and visuospatial abilities.	Neuropsychological tests scores are similar in the three groups except for Rey's figure copy that is better per­formed from subjects ChEIs plus TNP vs subjects treated only with ChEIs (p=0.02). The group treated only with ChEIs expressed, at follow-up, less depressive symptoms than at baseline (mean GDS 4.4±2.6 vs 3.5±2.7; p<0.05). The group treated with ChEIs and TNP improved in cognitive areas such as episodic memory (mean short story 7.5±2.6 vs 11.0±3.5; p<0.01) and abstract reasoning (mean Raven's colored matrices 24.2±3.1 vs 26.6±4.2; p<0.02) and in behavioural disturbances (mean NPI 18.7±7.9 vs 10.7±7.0/ p<0.016). Particularly it has been observed a reduction of depression (p<0.05) evaluated with NPI. The combined ChEIs and TPN treatment improves also depressive symptoms evaluated with GDS (mean 3.5±2.1 vs 2.2±1.3; p<0.02).	

Levine et al.^31^ and Winocur et al.^32^ described the cognitive rehabilitation of elderly subjects with memory complaints over a period of twelve weeks. Focusing on a modular training program, two groups of elderly subjects (Group 1 N=29 and Group 2 N=20, average age 79 years, SD=2.4 years) with memory complaints received a neuropsychological rehabilitation program based on three modules. Three subjects were excluded due to technical reasons. The first module, named Memory Skills Training focused on how to use the external and internal strategies to learn, retain and retrieve the information including diaries and notes. The second, the Modified Goal Management Training – GMT^33^ trained subjects to plan and organize fictitious and real life activities breaking down complex tasks into small and simple steps while monitoring the outcome. The third, Psychosocial Training aimed at psychosocial well-being enhancing self-confidence and trust in their cognitive abilities. Each module lasted 4 weeks and the two groups of elderly subjects received the 3 modules in separate periods. The results indicated an improvement in real-life tasks and on a self-reported questionnaire (DEX) after the three-module program in both groups. There was also evidence of some generalization to practical situations. The limitation of these studies included the reduced sample, the need to better refine the program of training and adaptation of the protocol to address other cognitive deficits.^31,32^

Talassi et al. (2007)^34^ assessed the effectiveness of a structured cognitive rehabilitation program based on computer cognitive training of neuropsychological and behavioral difficulties and functional status in a group of elderly subjects with MCI (N=30) and mild dementia (MD, N=24). This program was compared to a control condition where subjects received physical rehabilitation, occupational therapy and behavioral training. The experimental cognitive rehabilitation program consisted of three activities: computerized cognitive training (CCT), occupational therapy (OT) and behavioral training (BT). The CCT used the TNP software^24,25^ to stimulate each cognitive function addressing groups of exercises; the OT stimulated the patients to reproduce or simulate basic activities of daily living and the BT treated mood disorders with behavioral therapy. The control group received either physical rehabilitation (PR), OT or BT. Each group had a 30-40 minute session held for every activity, 4-days a week for a 3-week period. The results indicated that systematic rehabilitation with the computer training program showed an improvement in constructive apraxia, visuospatial recall, functional status and mood in the MCI group. The group with MD also showed an improvement on global cognitive function assessed by the MMSE and mood. The control group showed no significant improvement.

Cipriani et al.^26^ investigated the outcome of a computer-based cognitive training program in patients with MCI (N=10), Alzheimer’s disease (AD, N=10) and multiple system atrophy (MSA, N=3). Each patient attended two training programs with an interval of 4-8 weeks. The sessions lasted for 13-45 minutes over 4 days per week during a 4-week-period. The computer program was the same as mentioned in the previous study by Talassi et al.^34^ They used a modified version of the NPT software which aimed to stimulate attention, memory, perception, visuospatial abilities, language and non-verbal intelligence. Patients were assessed before and after 3 months at the end of the second training program. The MCI group improved on memory test score and the AD group improved on verbal fluency, executive functions and on MMSE scores. The MSA group did not show any changes in scores before and after treatment. The data from this research suggest that TNP can be used in specific groups of patients with memory impairment although no generalization effects were described.

Akhtar et al.^35^ adopted the “Errorless learning”– EL approach, i.e. learning without making mistakes, in patients with MCI (N=16). The results indicated that this technique was an effective tool showing significant improvements in the recall of a list of words in comparison to Errorful learning (EF). An important aspect considered in this study was the fact that in the group with memory impairment, the rehabilitation allowed for effective learning of new information by deploying this paradigm (EL).

Ball et al. (2007)^36^ combined data from six studies using a specified protocol of speed of processing training in order to evaluate training gains. The six studies included 2,039 elderly subjects (mean age=73.94, SD=5.96) with or without speed of processing deficits and all of them investigated the impact of completion of a computerized speed of processing training protocol on the cognitive and everyday abilities of older adults. The results suggested that training produced immediate improvements across all subtests and performance on instrumental activities and driving especially in those with speed of processing deficits. In addition, age and education had little to no impact on training gains and the participants maintained these gains for at least 2 years.

In another study, Ball et al. (2002)^37^ investigated whether cognitive training interventions improved mental abilities and daily functioning in elderly, independent-living adults with memory complaints. The 2832 participants aged 65 to 94 years’ old and were recruited from six metropolitan areas in the United States. Each intervention improved the targeted cognitive ability compared with baseline over two years. Three distinct cognitive interventions were used: memory, reasoning and speed training. Participants received instructions regarding strategies or mnemonic rule, and individual exercises or group feedback about their performance. They were trained to organize word lists with images and mental associations in order to recall them. The exercises involved laboratory memory tasks (e.g. recalling a list of nouns) as well as memory tasks related to cognitive activities of everyday life (e.g. remembering a shopping list). Reasoning training focused on the ability to solve problems in a serial pattern. Speed-of-processing training focused on visual search skills and ability to identify and locate visual information quickly in a divided-attention format. The results suggested that the cognitive training interventions improved targeted cognitive abilities in an effective way. However, no training effects on everyday functioning were detected after two years.

Subjects diagnosed with MCI who were submitted to neuropsychological training associated to cholinesterase inhibitors (ChEIs),^38^ achieved in the long term, cognitive improvement in memory, abstract reasoning and humor. It was evident that the combined treatment produced potencializing effects in the reduction of memory deterioration and in the delay of conversion from MCI to AD. Since there was no longer-term follow-up in this study, it was not possible to observe the presence of deterioration or whether the effect of improvement was associated with the training or resulted from a possible synergy between the cholinesterase inhibitors and neuropsychological training.

## Discussion

The aim of this study was to review the benefits of cognitive rehabilitation techniques in patients with cognitive complaints and MCI.

Cognitive training has been based on theories that support the use of specific techniques in order to optimize cognitive functioning (e.g. mnemonic techniques) in individuals with cognitive impairments and MCI, and to slow the rate of cognitive decline and handicap in daily life activities.

The main findings of the studies included in this review suggest that neuropsychological rehabilitation is recommended for patients with cognitive complaints, MCI and pre-clinical dementia which employ the Errorless Learning strategy.

Clare et al.^39,40^ suggested that awareness by the participants in terms of their potential and limitations leads to significant benefits from the rehabilitation techniques.

Another issue raised by the studies is the fact that programs of cognitive rehabilitation do not necessarily provide generalization from a controlled environment into everyday life situations. Even if there were transfer of the beneficial effects to daily activities, these would be restricted to a limited number of tasks. There are only few Class I studies in the literature on cognitive rehabilitation of these subjects and one of the reasons is the fact that there are few techniques including computerized training programs available, and also because this is a relatively new area in comparison to the rehabilitation of patients with AD.

Nevertheless, the implementation of rehabilitation programs with this population should be based on knowledge of the disease process, identifying the preserved and impaired abilities and handicaps. Knowledge of the most appropriate techniques to be applied for specific difficulties is also key, as is focusing on an errorless learning approach and always considering the carers as an integral part of the treatment receiving information and emotional support. It is also necessary to identify the consequences of cognitive impairment to daily life activities, since the goal of neuropsychological rehabilitation is not an improvement in the performance of cognitive tests but rather an improvement in quality of life of the patients, family members and caregivers.
